# HVEM/HIF-1α promoted proliferation and inhibited apoptosis of ovarian cancer cells under hypoxic microenvironment conditions

**DOI:** 10.1186/s13048-020-00646-3

**Published:** 2020-04-20

**Authors:** Liyan Duan, Jie Tao, Xiaoqian Yang, Lei Ye, Yueqian Wu, Qizhi He, Yingchun Duan, Li Chen, Jianlong Zhu

**Affiliations:** 1 Shanghai First Maternity and Infant Hospital, Tongji University School of Medicine, Shanghai, China; 2 Department of Gynecology and Obstetrics, Shanghai Pudong Hospital, Fudan University School of Medicine, No. 2800 Gongwei Road, Pudong New Area, Shanghai, 201399 China

**Keywords:** HVEM, Ovarian cancer, HIF-1α, Hypoxic microenvironment

## Abstract

**Background:**

Our previous studies showed the expression of herpes virus entry mediator (HVEM) is high in ovarian cancer samples and correlated to the patient clinic pathological features. As we all know, the hypoxic environment is the main feature of tumor. In this work, we explored the role of HVEM in hypoxic ovarian cancer cells and its effects on HIF-1α, a transcription factor responding to hypoxia.

**Methods:**

The expression of HVEM, HIF-1α and apoptosis-related genes was detected by qRT-PCR and western blot. The proliferation and apoptosis of the ovarian cancer cells were determined with the Cell Counting Kit-8 assay and AnnexinV-FITC/PI-stained flow cytometry assay, respectively.

**Results:**

The expression of HVEM was positively correlated to that of HIF-1α. The expression of HVEM and HIF-1α under hypoxic conditions was higher than that under normoxic conditions, which suggested that the level of HVEM and HIF-1α correlates with prolonged periods of hypoxia in ovarian cancer. The overexpression of HVEM promoted cell proliferation and inhibited cell apoptosis under hypoxic condition. HVEM overexpression elevated the expression of HIF-1α and Bcl-2 (anti-apoptotic protein), and reduced the expression of Bax (pro-apoptotic protein). In addition, overexpression of HVEM activated the AKT/mTOR signaling. Moreover, knockdown of HVEM had the completely opposite effects.

**Conclusion:**

These data indicated that HVEM signaling might promote HIF-1α activity via AKT/mTOR signaling pathway and thus to regulate tumor growth in ovarian cancer under the hypoxic conditions. Furthermore, these findings indicate that this molecular mechanism could represent a therapeutic target for ovarian cancer.

## Introduction

Ovarian cancer is the most commonly diagnosed malignant gynecological tumor and the primary cause of tumor-related mortality for genital system. Total 238,700 new cases and 151,900 deaths in situ of ovary were estimated worldwide in 2012; they accounted for 3.6% of all estimated new cases and 4.3% of all estimated deaths among female [1]. In United States, total 22,240 new cases and 14,070 deaths of ovary in situ, accounting for 2.5% of all estimated new cases and 4.9% of all estimated deaths among female, were estimated in 2018 [2]. Ovarian cancer was commonly treated with surgery, chemotherapy, and radiotherapy, which can alleviate some symptoms. However, these treatments seriously affect the quality of patient life and the recurrence rate is still high. Moreover, although much effort has been expended in the diagnosis and therapy of ovarian cancer, the 5 year survival rate is < 40% in patients [3]. The poor prognoses of ovarian cancer patients are related to chemotherapy resistance and tumor metastasis [4]. In recent years, numerous preclinical studies and clinical trials have shown that the microenvironment of the tumor is crucial to the malignant biological characteristics of these tumors [5]. The main feature of the tumor microenvironment is tissue anoxia [6]. Therefore, new therapeutic strategies that considered the hypoxic microenvironment are of great significance for the therapy of ovarian cancer.

Hypoxia-inducible factor 1 (HIF-1), consisting of HIF-1α and HIF-1β, is a heterodimeric protein. The expression of transcription factor HIF-1α is induced by a hypoxic environment and is maintained at a high level under hypoxic conditions [7, 8]. HIF-1α alters the metabolism of tumor cells, causes the release of the proangiogenic factors vascular endothelial growth factor (VEGF), N-myc downstream regulated 1 (NDRG1) and Glucose transporter 1 (GLUT1), promotes cell proliferation and inhibits cell apoptosis. Therefore, cancer cells can adapt and tolerate a hypoxic environment and even proliferate infinitely [9–11]. HIF-1α can be used to predict tumor progression and the overexpression HIF-1α is correlated to poor survival in the clinical context [12]. Tumor cells also display stem-cell-like properties, allowing their infinite proliferation under hypoxic conditions [13]. A recent study demonstrated that adrenomedullin promoted tumor angiogenesis by up-regulating the level of VEGF and HIF-1α in ovarian cancer [14], which suggested the importance of HIF-1α in the treatment of ovarian cancer.

Herpes virus entry mediator (HVEM) regulates the cellular entrance of Herpes simplex virus, and is also known as tumor necrosis factor receptor super family member 14 [15]. A clinical study demonstrated that HVEM was closely related to tumor development and immune evasion, which implied that it might be a new prognostic marker and underlying target for human tumors [16]. In our previous finding, we showed that the expression of HVEM was significantly high in ovarian cancer tissues and was closely correlated to the clinical pathological features of the patient, including the clinical stage (FIGO2013), lymph-node metastasis and recurrence [17]. However, the overexpression of HVEM had no effects on the cell multiplication in normoxic ovarian cancer cells [18]. In this work, we investigated the role of HVEM in hypoxic ovarian cancer cells and the relationship between HVEM and HIF-1α by overexpressing and silencing HVEM. This study demonstrates the potential utility of HVEM in novel therapeutic strategies for ovarian cancer.

## Results

### HVEM co-expressed with HIF-1α in patients with ovarian cancer

**Fig. 1:**
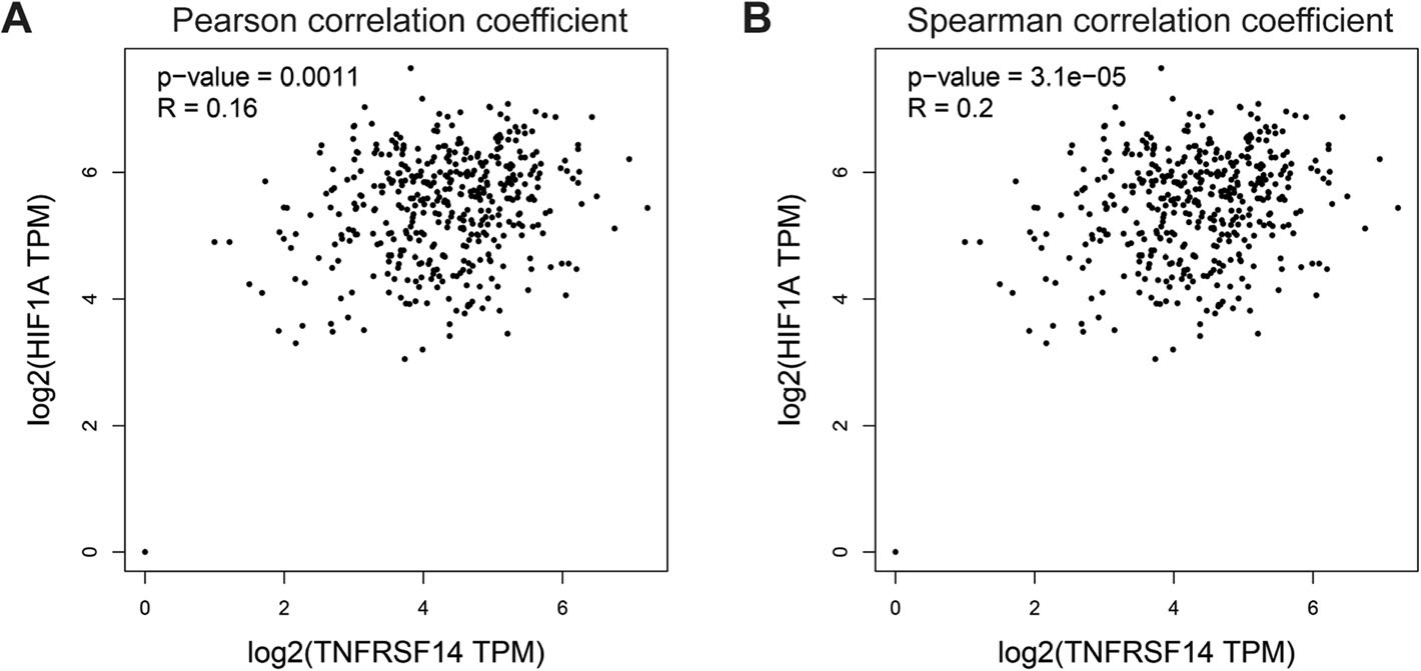
HVEM co-expressed with HIF-1α in patients with ovarian cancer. Expression correlation analysis of HEVM and HIF-1α in ovarian cancer tissues by Pearson (**a**) and Spearman (**b**)

In our previous study, HVEM was found highly expressed in the ovarian cancer tissues compared with normal tissues [18]. Moreover, HVEM was correlated to TNM stage, lymph node metastasis and recurrence in ovarian serous adenocarcinoma [17]. Here, we analyzed the expression correlation of HVEM and HIF-1α using the online analysis tool Gene Expression Profiling Interactive Analysis [19]. The expression of HVEM was positively associated with that of HIF-1α (Fig. 1a and b) (Pearson R = 0.16, *P* = 0.001; Spearman R = 0.2, *P* = 3.1e-05). Thus, hypoxic microenvironment might mediate the effects of HVEM in promoting the development of ovarian cancer.

### Hypoxia enhanced the expression of HIF-1α and HVEM

**Fig. 2:**
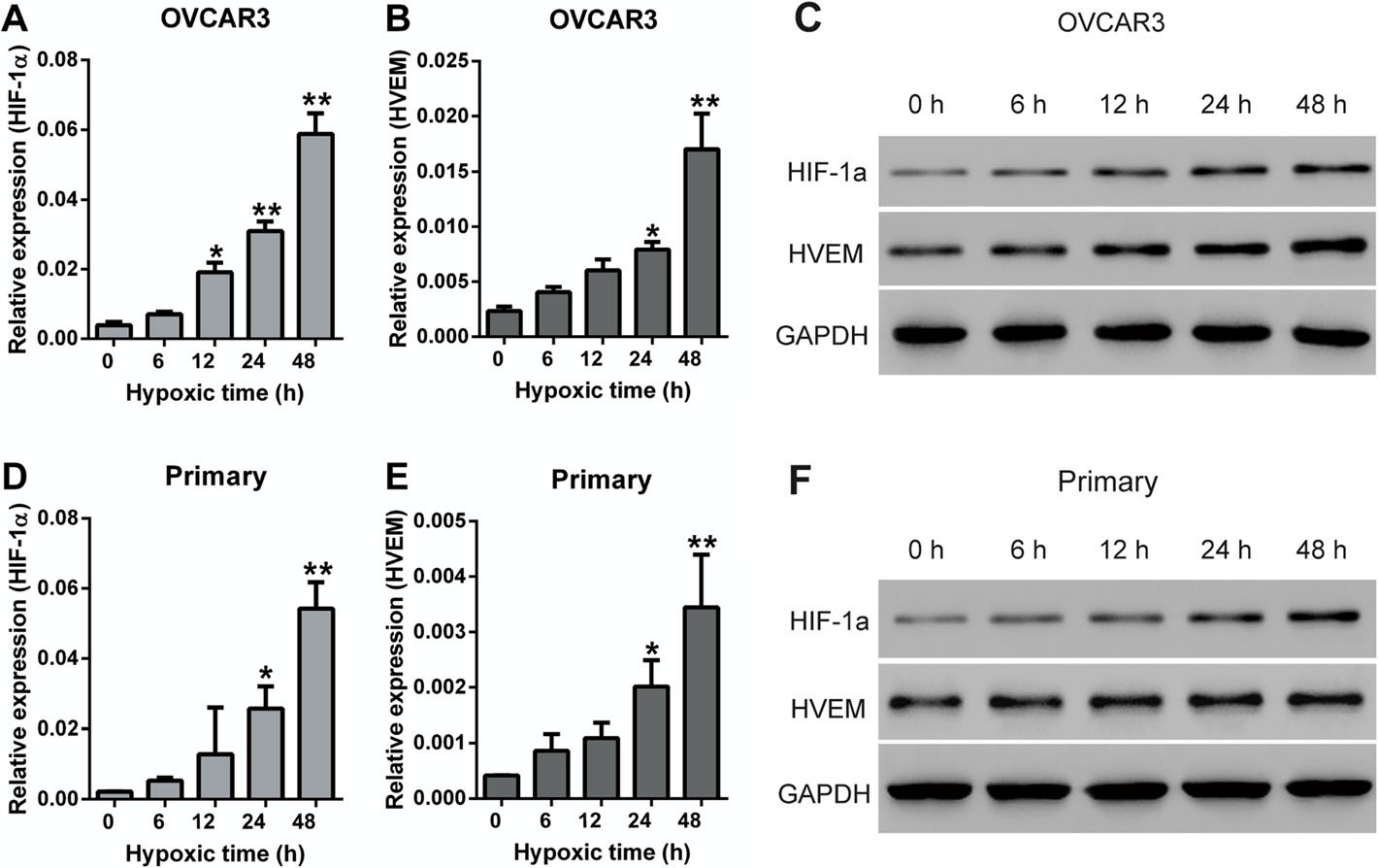
The expression of HIF-1α and HEVM in OVCAR3 cells and primary ovarian cancer cells under hypoxic conditions. The mRNA expression (**a** and **b**) and protein expression (C) of HIF-1α and HEVM in OVCAR3 cells under hypoxic conditions. The mRNA expression (**d** and **e**) and protein expression (**f**) of HIF-1α and HEVM in primary ovarian cancer cells under hypoxic conditions. Primary: primary ovarian cancer cells. ^*^P < 0.05, ^**^P < 0.001 vs. hypoxia for 0 h; *n* = 3

The primary ovarian cancer cells were isolated from the ovarian cancer tissues and identified by immunofluorescence staining for pan-cytokeratin (CK) and calretinin (CR). Almost all ovarian cancer cells were CK-positive, and all cells were CR-negative (Fig. S1), indicating the isolated cells are truly ovarian cancer cells. To validate the function of HVEM in ovarian cancer under hypoxic microenvironment, OVCAR3 cells and primary ovarian cancer cells were cultured under hypoxic conditions, which mimicked a more realistic tumor environment, and the expression of HIF-1α and HVEM in the hypoxic ovarian cancer cells was detected by qRT-PCR and western blot. Hypoxia induced elevated mRNA (Fig. 2a, b, d and e) and protein ((Fig. 2c and f) expression of HIF-1α and HVEM. Moreover, both the expression of HIF-1α and HVEM was gradually increased over time. After hypoxia for 12 h in OVCAR3 cells and hypoxia for 24 h in primary cells, the expression of HIF-1α significantly enhanced. However, the expression of HVEM markedly increased after hypoxia for 24 h in both OVCAR3 cells and primary cells. Therefore, hypoxia for 24 h was used in the following study.

### Identification of HVEM knockdown and overexpression efficiency

**Fig. 3:**
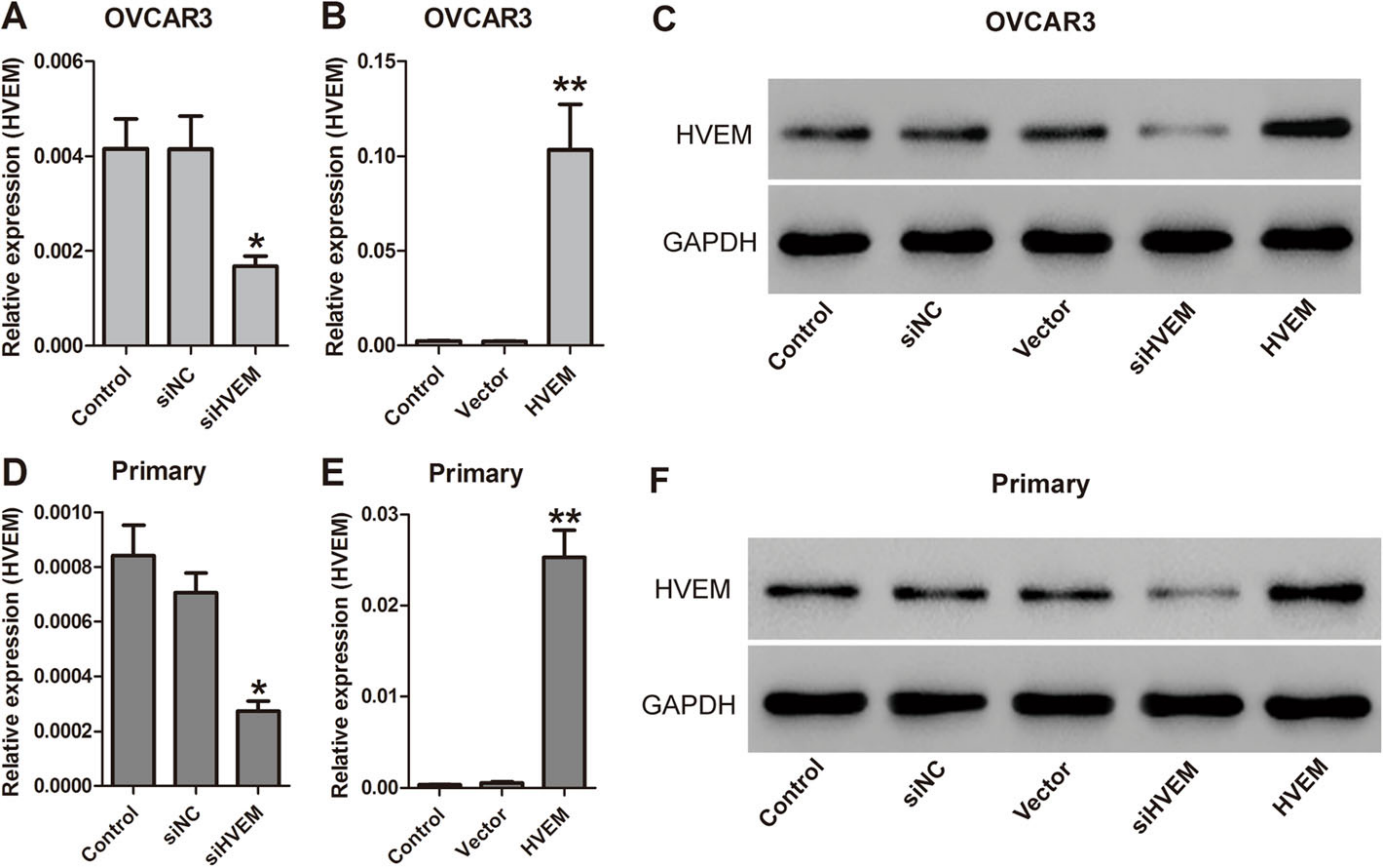
HVEM expression in OVCAR3 cells (**a**-**c**) and primary ovarian cancer cells (**d**-**f**) transfected with siHVEM or HVEM vector by qRT-PCR and Western blot. ^*^P < 0.05, ^**^P < 0.001, n = 3

To explore the function of HVEM in ovarian cancer, OVCAR3 cells and primary ovarian cancer cells were transfected with a small interfering RNA targeting HVEM (siHVEM) or pCDNA-HVEM (HVEM) to interfere the expression of HVEM. The knockdown and overexpression efficiency was identified by qRT-PCR and western blot. OVCAR3 cells transfected with siHVEM had significantly decreased expression of HVEM (*P* = 0.004), while the cells transfected with HVEM had obviously increased expression of HVEM (*P* = 0.000) (Fig. 3a-c). The siHVEM and HVEM vector also had similar effects on HVEM expression in primary ovarian cancer cells (Fig. 3d-f). All these results suggest that the expression of HVEM in both OVCAR3 cells and primary ovarian cancer cells are effectively silenced or overexpressed.

### Effects of HVEM on cell proliferation of hypoxic ovarian cancer cells

**Fig. 4:**
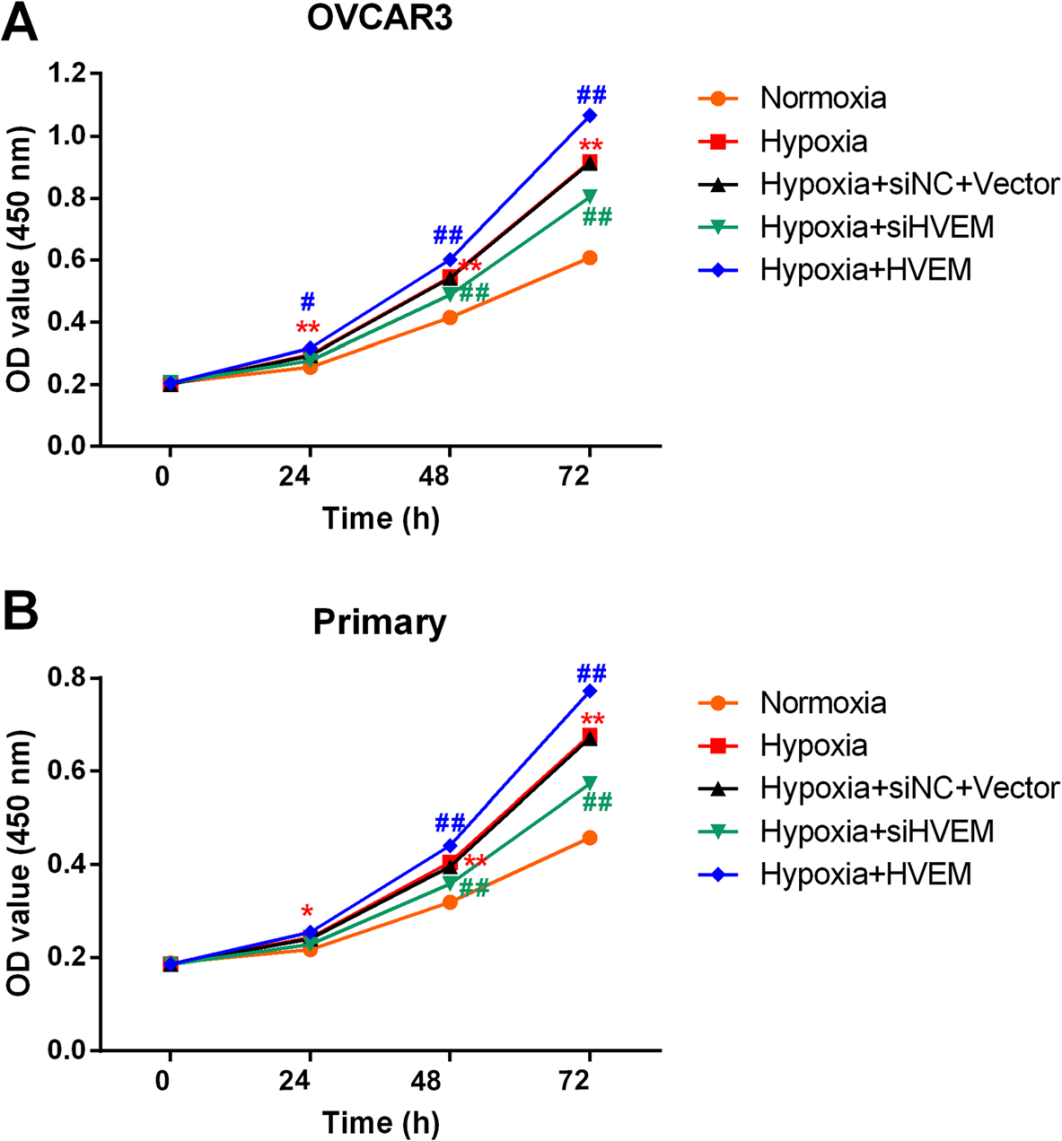
Effects of HVEM on the proliferation of OVCAR3 cells (**a**) and primary ovarian cancer cells (**b**) under hypoxic conditions. ^*^P < 0.05, ^**^P < 0.001 vs. Normoxia group; ^#^*P* < 0.05, ^##^*P* < 0.001 vs. Hypoxia group; n = 3

The cell proliferation was measured by CCK-8 assay. As shown in Fig. 4, the cell viability was higher in both hypoxic OVCAR3 cells and hypoxic primary cells compared with that in normoxic OVCAR3 cells and primary cells. Knockdown of HVEM inhibited hypoxia-induced cell proliferation, while overexpression of HVEM promoted hypoxia-induced cell proliferation further.

### Effects of HVEM on cell apoptosis of hypoxic ovarian cancer cells

**Fig. 5:**
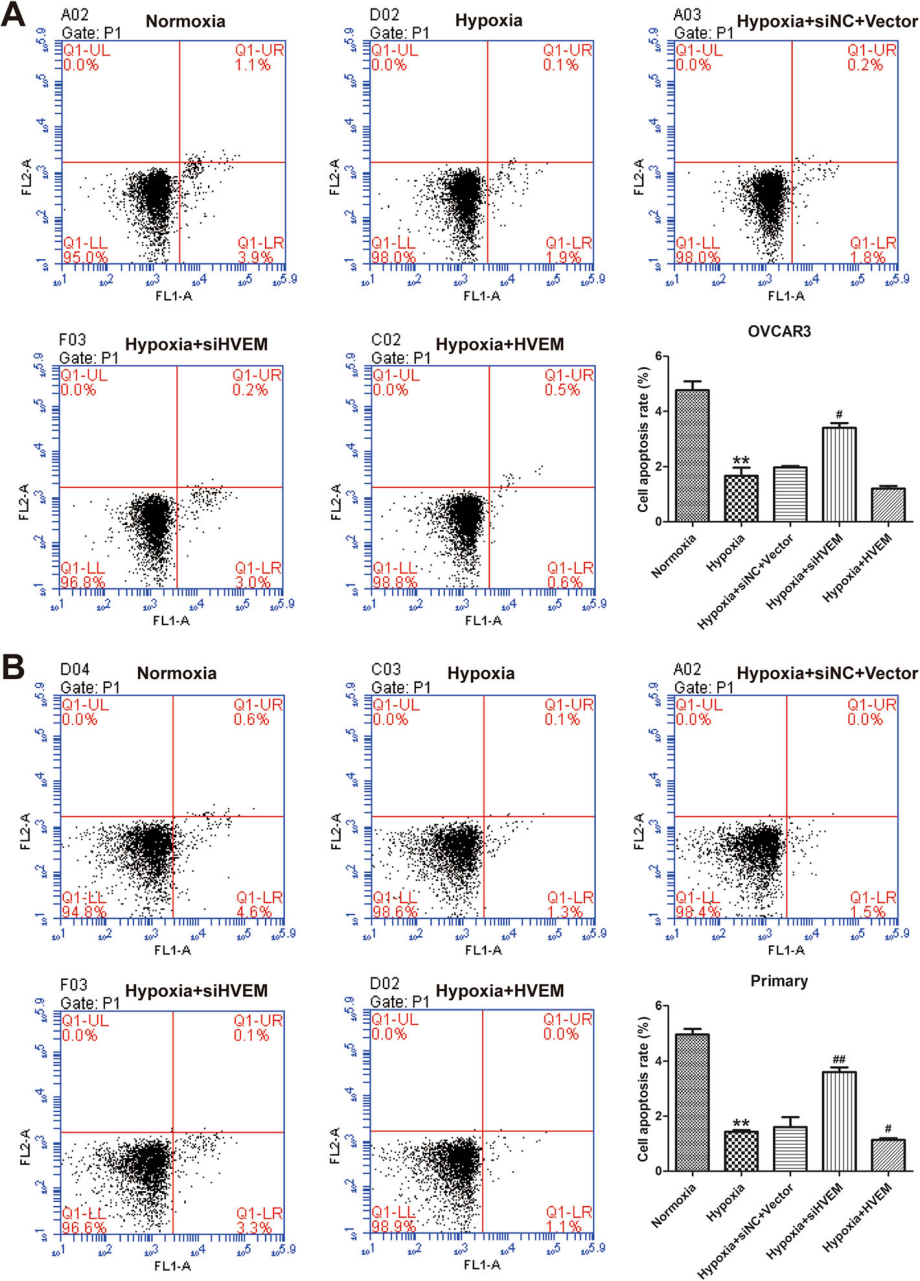
Effects of HVEM on the apoptosis of OVCAR3 cells (**a**) and primary ovarian cancer cells (**b**) under hypoxic conditions. ^**^P < 0.001 vs. Normoxia group; ^#^*P* < 0.05, ^##^*P* < 0.001 vs. Hypoxia group; n = 3

The cell apoptosis was detected by AnnexinV-FITC/PI-stained flow cytometry assay. Hypoxia suppressed the cell apoptosis of OVCAR3 cells (Fig. 5a) and primary cells (Fig. 5b). Knockdown of HVEM promoted cell apoptosis in hypoxic OVCAR3 cells and primary cells; while overexpression of HVEM inhibited cell apoptosis in hypoxic OVCAR3 cells and primary cells further.

### Effects of HVEM on the expression of apoptosis-related proteins in hypoxic ovarian cancer cells

**Fig. 6:**
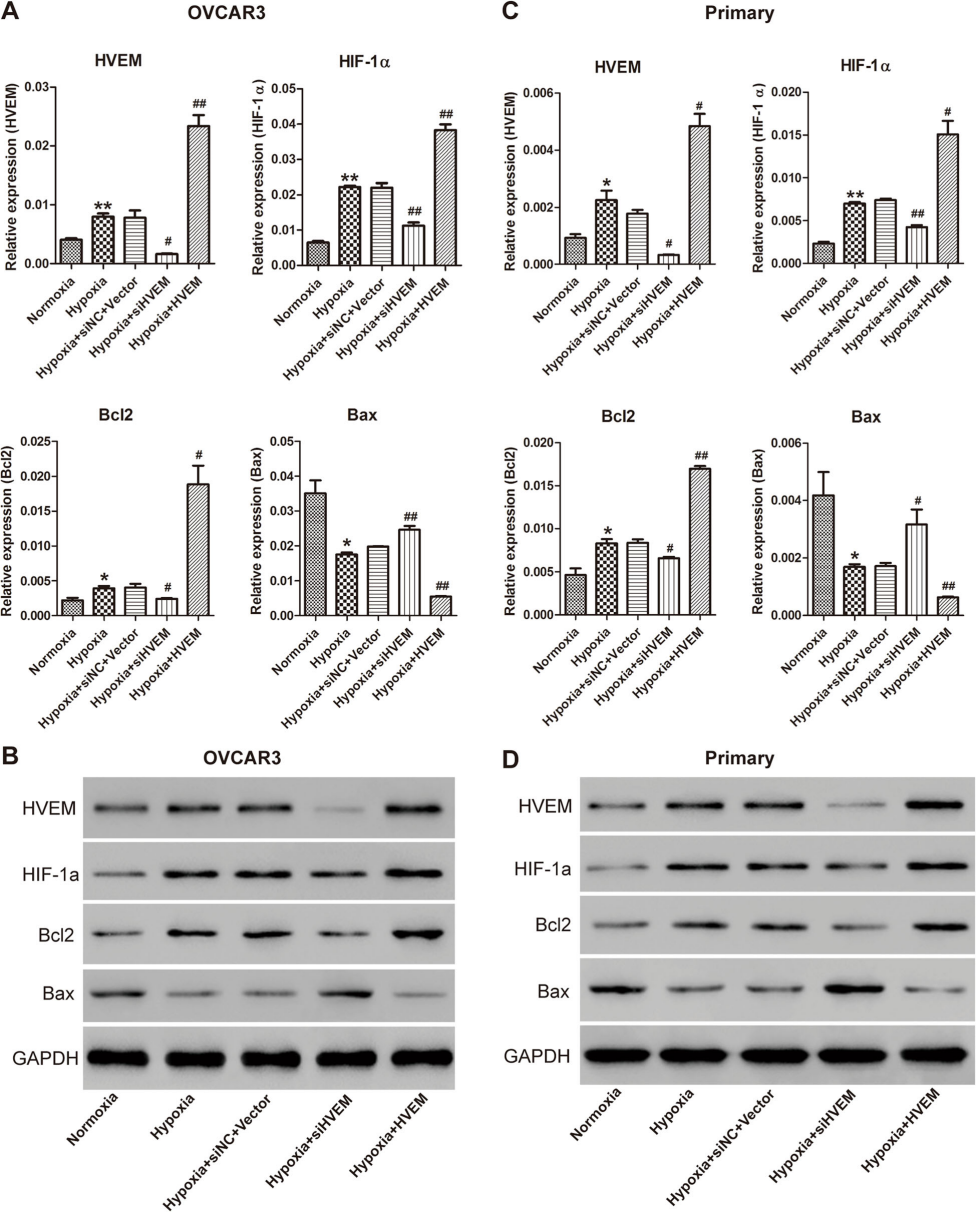
Effects of HVEM on the expression of HIF-1α and apoptosis-related protein in hypoxic OVCAR3 cells and hypoxic primary ovarian cancer cells. The mRNA (**a**) and protein (**b**) expression of HIF-1α and apoptosis-related protein in hypoxic OVCAR3 cells. The mRNA (**c**) and protein (**d**) expression of HIF-1α and apoptosis-related protein in hypoxic primary ovarian cancer cells. ^*^P < 0.05, ^**^P < 0.001 vs. Normoxia group; ^#^*P* < 0.05, ^##^*P* < 0.001 vs. Hypoxia group; n = 3

To further explore the underlying mechanism of HVEM in hypoxic ovarian cancer cells, the expression of HVEM, HIF-1α, Bcl-2 (anti-apoptotic protein) and Bax (pro-apoptotic protein) was determined by qRT-PCR and western blot. Knockdown of HVEM significantly reduced hypoxia-induced expression of HIF-1α in hypoxic OVCAR3 cells and primary ovarian cancer cells. As presented in Fig. 6, overexpression of HVEM markedly enhanced hypoxia-induced expression of HIF-1α in hypoxic OVCAR3 cells and primary ovarian cancer cells further. Moreover, knockdown of HVEM significantly decreased the expression of Bcl-2 and increased the expression of Bax in hypoxic OVCAR3 cells and primary ovarian cancer cells; while overexpression of HVEM had the opposite effects.

### Role of AKT/mTOR signaling pathway in the effects of HVEM in hypoxic ovarian cancer cells

**Fig. 7:**
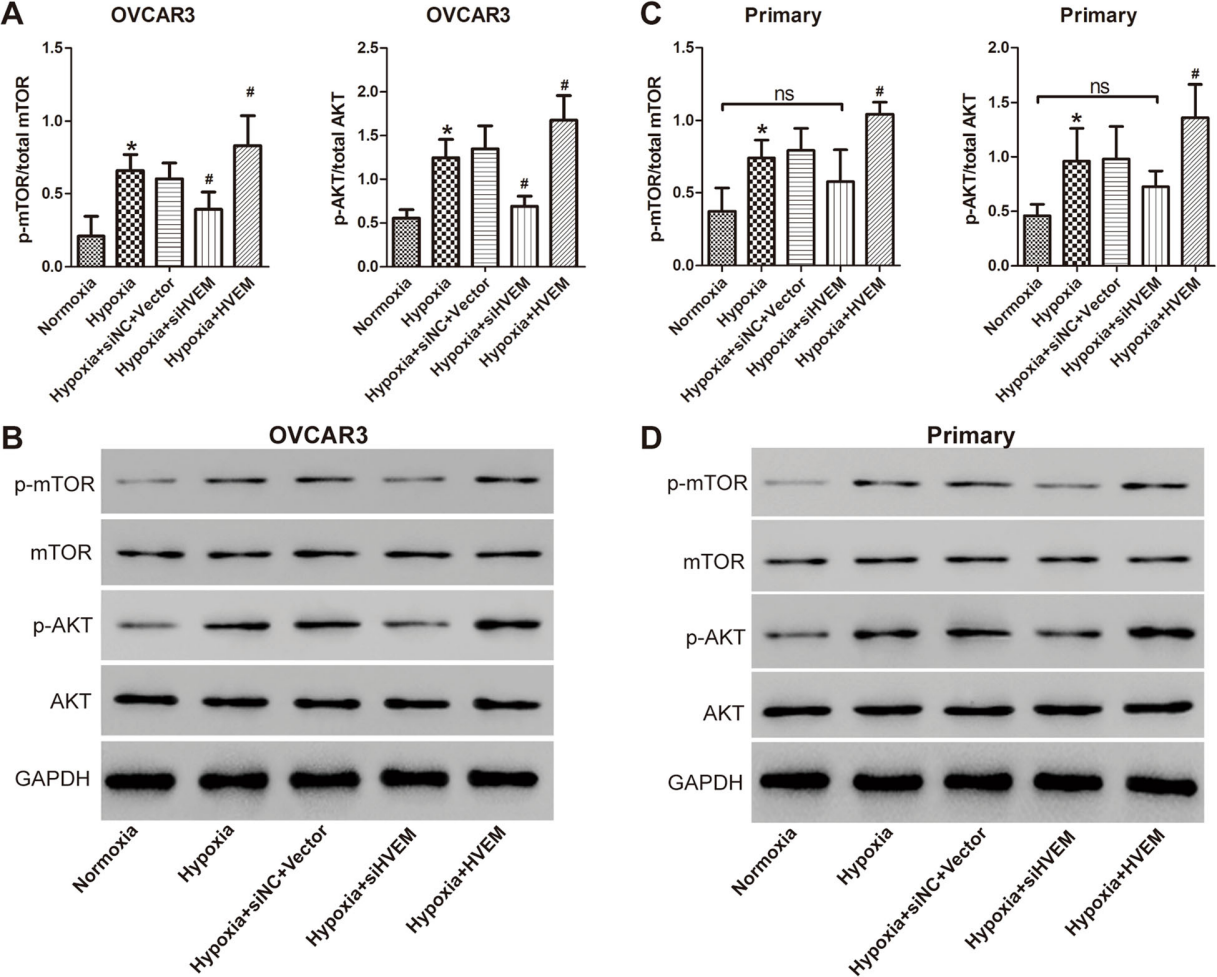
Role of AKT/mTOR signaling in the effects of HVEM in hypoxic OVCAR3 cells (**a** and **b**) and hypoxic primary ovarian cancer cells (**c** and **d**). ^*^P < 0.05 vs. Normoxia group; ^#^*P* < 0.05 vs. Hypoxia group; n = 3

It has been demonstrated that AKT/mTOR signaling pathway play a vital role in the regulation of HIF-1α in ovarian cancer [20, 21]. To investigate whether AKT/mTOR signaling pathway mediated the regulatory role between HVEM and HIF-1α, the expression of AKT/mTOR signaling pathway related protein was determined after intervening expression of HVEM by western blot. Hypoxia induced activation of AKT and mTOR in OVCAR3 cells (Fig. 7a and b). Knockdown of HVEM markedly blocked the signaling of AKT and mTOR in OVCAR3 cells, while overexpression of HVEM significantly promoted the activation of AKT and mTOR in OVCAR3 cells further. The activation of AKT and mTOR was also induced in hypoxic primary ovarian cancer cells (Fig. 7c and d). Silence of HVEM down-regulated the expression of the phosphorylated AKT and mTOR in primary ovarian cancer cells. The regulation was not statistically significant. However, notably, no statistically significant difference was found in the expression of phosphorylated AKT and mTOR between normoxia group and hypoxia+siHVEM group. Moreover, overexpression of HVEM significantly promoted the activation of AKT and mTOR in primary ovarian cancer cells further. All these findings indicated that AKT/mTOR signaling was implicated in the role of HVEM in hypoxic ovarian cancer.

## Discussion

The incidence of ovarian cancer has a progressive increase from 1999 to 2015 with annual percent changes (APC) of 1.8% in Korean [22]. It is well known that a lack of sufficient oxygen is a major characteristic of tumors, and these hypoxic conditions promote the epithelial–mesenchymal transition and tumor metastasis [23–25]. Therefore, identifying and tracking the markers under more realistic hypoxic environment should provide more information about the mechanisms of proliferation, apoptosis, invasion, and metastasis for ovarian cancer.

HIF-1α has been reported to play an important role in the metabolism and metastasis of cancer cells [26]. The expression of HIF-1α is regulated by multiple factors and HIF-1α highly expressed in many kinds of cancers, correlating with the proliferation, invasion and metastasis. Its high expression is associated with a poor prognosis [6, 27]. Numerous drugs inhibiting the activity of HIF-1α had been identified as anti-cancer agents [28–30]. HVEM was first identified in 1996 when screening for genes that mediate the entry of *Herpes simplex virus* into Chinese hamster ovary-K1 cells [15]. HVEM is the first member of the TNFR superfamily and expressed highly in many tissues, especially in those rich in lymphocytes, such as the spleen and lymph nodes. Our previous study found that HVEM expressed highly in ovarian cancer samples and associated with the patient clinicopathological features, including TNM staging, lymph node metastasis and recurrence [17]. In this study, the expression of HVEM was found positively correlated to that of HIF-1α in ovarian cancer. Therefore, we speculated that HVEM might exert its effects in the development of ovarian cancer via regulation of HIF-1α expression.

Moreover, in the present study, the expression of HIF-1α was gradually increased in hypoxic ovarian cancer cells when exposed to prolonged hypoxia. Notably, the expression of HVEM had the same trend as HIF-1α. Knockdown of HVEM markedly reduced the expression of HIF-1α; while overexpression of HEVM significantly increased the expression of HIF-1α. The results were consistent with the previous report that HVEM increased the activity of HIF-1α in vivo [31]. Additionally, exogenously expressing HVEM in primary ovarian cancer cells and OVCAR-3 cells markedly reduced the expression of Bax (pro-apoptotic protein) and significantly increased the expression of Bcl-2 (anti-apoptotic protein). However, knockdown of HVEM had the completely opposite impact. It has been reported that whether a cell lives or dies is mainly determined by the anti-apoptotic regulators Bcl-2 family and the apoptosis-promoting protein Bax [32].When the expression of Bcl-2 exceeds that of BAX, cells do not undergo apoptosis. However, when the expression of Bax is dominant, cells are susceptible to apoptosis in response to inducers [33]. Therefore, inhibiting the expression of HVEM should suppress the proliferation of cancer cells. Our data also indicate that when the expression of HVEM was silenced, the cell apoptosis rate increased significantly.

AKT/mTOR signaling pathway has been known to play a vital role in the manipulation of HIF-1α in ovarian cancer [20, 21]. In the present study, knockdown of HVEM markedly blocked the activation of AKT and mTOR, while overexpression of HVEM significantly promoted the signaling of AKT and mTOR in OVCAR3 cells and primary ovarian cancer cells further. Therefore, HVEM might regulate the expression level of HIF-1α via AKT/mTOR signaling pathway. However, the specific mechanism is still needed to be studied further.

In summary, the current study partially explained the relationship between the HEVM and HIF-1α under the hypoxic conditions. We observed that the HVEM promoted the expression of HIF-1α. To some extent, HVEM up-regulated the expression level of Bcl-2 and down-regulated that of Bax, which mediated the cancer cell apoptosis. Therefore, we assume that HVEM might lead to the development of ovarian cancer by regulating the expression of HIF-1α. These findings suggest a possible novel targeted therapeutic strategy for ovarian cancer. The effect of HVEM- HIF-1α axis in ovarian cancer is not fully elaborated. There are many works needed to do in the future work.

## Materials and methods

### Isolation and identification of primary ovarian cancer cells and cells culture

The experiments were approved by the Ethical Committee of Shanghai First Maternity and Infant Hospital, Tongji University School of Medicine and performed according to the Helsinki Declaration. The ovarian cancer tissues were obtained from Shanghai First Maternity and Infant Hospital, Tongji University School of Medicine. After surgery, small pieces of tumor tissue (0.3–0.8 cm) were placed in PBS containing penicillin and streptomycin and rotated for 1 h. After washing with PBS, the specimens were minced into 2 mm fragments and incubated with collagenase type II (800 u/ml; Sigma-Aldrich) at 37 °C for 30 min. Then, the cells were disaggregated by disruption and then filtered using a 100 μm cell strainer. We incubated the single primary ovarian cancer cells with anti-CD31- and anti-CD45-conjugated microbeads (Miltenyi Biotec, Auburn, CA, USA) with 30 min of rotation at 4 °C to remove the endothelial and hematopoietic cells. Then, the cells were collected and detected by immunofluorescence staining for pan-cytokeratin (CK) and calretinin (CR). The primary antibodies anti-pan-cytokeratin (sc-81,714, Santa Cruz Biotechnology, USA) and anti-calretinin (ab92341, Abcam, USA), and the secondary antibodies Alexa Fluor 488-labeled anti-rabbit IgG (A0423, Beyotime, JiangSu, China) and anti-mouse IgG (A0428, Beyotime, JiangSu, China) were applied. The nuclei were dyed with 4′, 6-diamidino-2-phenylinedole (DAPI, C1002, Beyotime, JiangSu, China).

OVCAR3 cells were bought from the Shanghai Institutes for Biological Sciences Cell Bank (Shanghai, China). The primary ovarian cancer cells and OVCAR3 cells were cultured in RPMI-1640 medium containing 1% antibiotics and 10% fetal bovine serum (Hyclone, Logan, UT, USA) in an incubator at 37 °C with 5% CO_2_ and 21% O_2_ (normoxia), or 5% CO_2_ and 1% O_2_ (hypoxia).

### Quantitative real–time polymerase chain reaction (qRT-PCR)

The RNA was extracted with TRIzol method. The cDNA was synthesized using the Prime Script RT Reagent Kit (Takara, Kyoto, Japan). PCR reactions were performed in triplicate. The primer sequences were showed in Table 1. PCR was carried out at 95 °C for 10 min and then 10 s denaturation at 95 °C and 1 min annealing at 60 °C for 40 cycles. The mRNA level of the target genes was normalized to GAPDH using the 2^-ΔΔCq^ method [34].

**Table 1:** Primer sequences for qRT-PCR

Gene	NCBI accession number	Primer sequences (5′ -3′)	Product length (bp)
GADPH	NM_001256799.2	F: AATCCCATCACCATCTTC	218
		R: AGGCTGTTGTCATACTTC	
HIF-1α	NM_001243084.1	F: TTCCAGCAGACTCAAATA	285
		R: ACTCAAAGCGACAGATAA	
HVEM	NM_001297605.1	F:ACTGCTCCAGGACAGAGAA	251
		R: ACGGAGACGATCACCTTGA	
Bcl2	NM_000633.2	F:TCCCTCGCTGCACAAATAC R:TGGAAGGCCACATCTGAAC	225
Bax	NM_001256799.2	F:AATCCCATCACCATCTTC R: AGGCTGTTGTCATACTTC	218

### Western blot

The cells were placed in the radioimmune precipitation assay buffer and then the the cellular proteins were separated with SDS-15% polyacrylamide gel electrophoresis under denaturing conditions. Then, after blocked with 5% skim milk, the nitrocellulose membrane transferred with proteins were incubated with primary antibodies against HVEM (1:500; Ab62462, Abcam, USA), HIF-1 α (1:500; Ab113642, Abcam), Bcl-2 (1:400; Sc-492, Santa Cruz Biotechnology), Bax (1:300; Sc-493, Santa Cruz Biotechnology), AKT [1:1000; #9272, Cell Signaling Technology (CST)], p-AKT (1:1000; #9271, CST), mTOR (1:1000; #2972, CST), p-mTOR (1:1000; #2971; CST) and GAPDH antibodies (1:2000; #5174, CST, Danvers, MA, USA), followed by a horseradish-peroxidase-conjugated secondary antibody (Beyotime, Shanghai, China). The signals were determined by an enhanced chemiluminescence system (Bio- Rad). GAPDH was selected as the internal control.

### Cell counting Kit-8 (CCK-8) assay

The cells were planted in 96-well plates (3 × 10^3^ cells per well) and cultured overnight. After 0, 24, 48 or 72 h of incubation, 10% CCK-8 solution (100 μl) was added into each well. The optical density at 450 nm (OD450) was then detected with a microplate reader (Labsystems, Helsinki, Finland).

### Cell apoptosis analyses

After 48 h of culture, the cells were collected and washed with PBS. Trypsin–EDTA solution was then sucked and added into the cell culture fluid with slight mixing. After 5 min of centrifugation, the upper supernatant was threw away and the cells were collected. We centrifuged 1 × 10^5^ cells suspended in 1000 ml for 5 min, and discarded the supernatant. Then, the cells were stained with propidium iodide (20 μg/ml; Sigma-Aldrich) and RNase (100 μg/mL; Sigma-Aldrich) in the dark for 20 min at 4 °C. An AnnexinV-FITC/PI apoptosis detection kit (Beyotime, JiangSu, China) was performed to stain the cells. Cell apoptosis was then determined with a flow cytometer.

### Statistical analysis

The data were presented as means ± standard deviations (SD) and analyzed with SPSS 22.0. The difference among groups was performed with one-way analysis of variance, followed by Tukey test. *P* < 0.05 was considered statistically significant.

## Supplementary information

**Additional file 1 Figure S1**. Immunofluorescence staining for pan-cytokeratin (CK) and calretinin (CR) in the isolated primary ovarian cancer cells.

## Data Availability

The datasets used or analyzed during the current study are available from the corresponding author on reasonable request.

## Abbreviations

HVEM: Herpes virus entry mediator
VEGF: Vascular endothelial growth factor
