# Supplementary figures and images for: HVEM/HIF-1α promoted proliferation and inhibited apoptosis of ovarian cancer cells under hypoxic microenvironment conditions
Source: J Ovarian Res. 2020 Apr 20;13:40. doi: 10.1186/s13048-020-00646-3 (PMC7168979; doi:10.1186/s13048-020-00646-3)

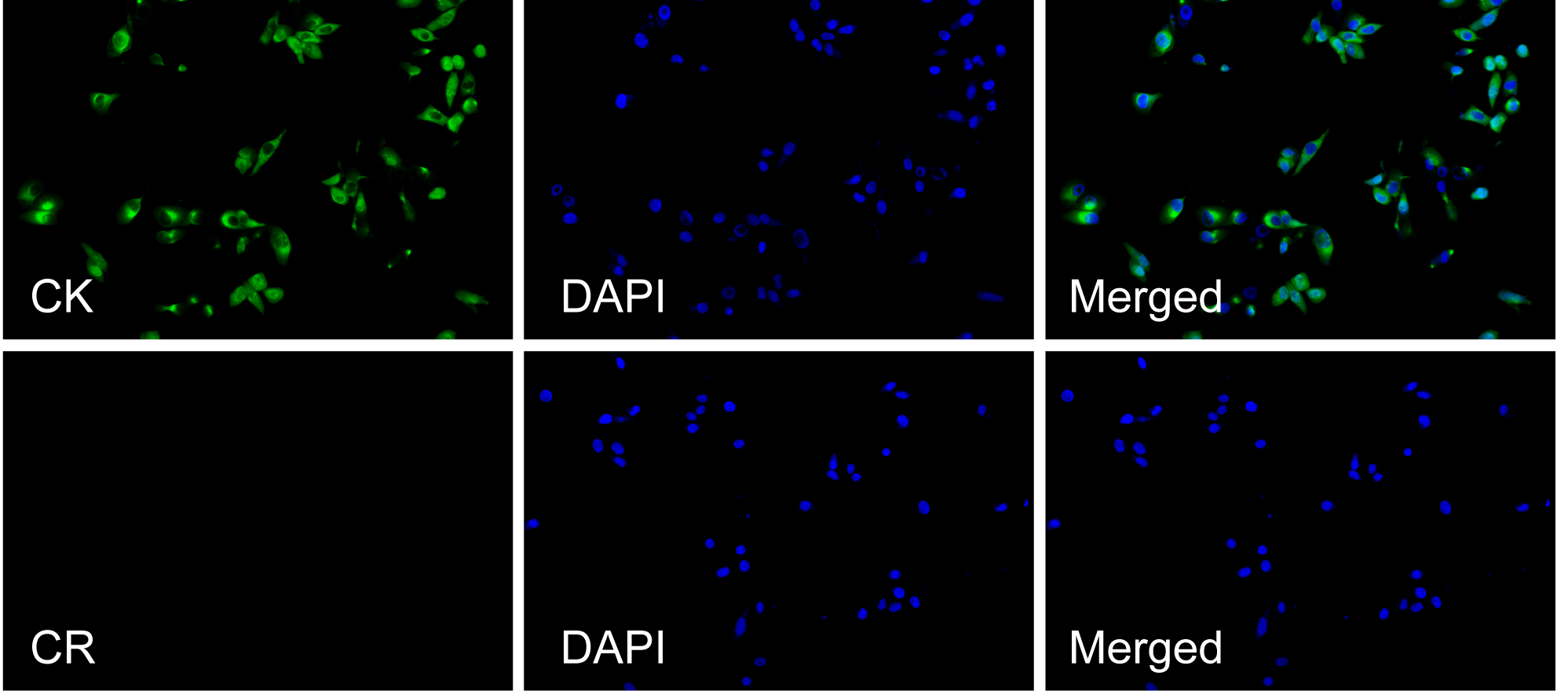

Supplement: Supplementary file 1 — Additional file 1 Figure S1. Immunofluorescence staining for pan-cytokeratin (CK) and calretinin (CR) in the isolated primary ovarian cancer cells. [file 13048_2020_646_MOESM1_ESM.tif]
